# Gram-negative multidrug-resistant organisms were dominant in neurorehabilitation ward patients in a general hospital in southwest China

**DOI:** 10.1038/s41598-022-15397-y

**Published:** 2022-06-30

**Authors:** Wei Jiang, Lang Li, Siyang Wen, Yunling Song, Lehua yu, Botao Tan

**Affiliations:** 1 Department of Rehabilitation Medicine, The Second Affiliated Hospital of Chongqing Medical University, Chongqing, 400010 China; 2 Department of Laboratory Medicine, The Second Affiliated Hospital of Chongqing Medical University, Chongqing, 400010 China

**Keywords:** Clinical microbiology, Risk factors, Health care, Rehabilitation

## Abstract

This study aimed to investigate the prevalence of and risk factors for multidrug-resistant organism (MDRO) infection in the rehabilitation ward of a general hospital in Southwest China. We analyzed rehabilitation patients with nosocomial infections caused by MDROs from June 2016 to June 2020. MDRO infection pathogens and associated antibiotic resistance were calculated. Possible risk factors for MDRO-related infection in the neurorehabilitation ward were analyzed using chi-square, and logistic regression. A total of 112 strains of MDRO were found positive from 96 patients. The MDRO test-positive rate was 16.70% (96/575). Ninety-five MDRO strains were detected in sputum, of which 84.82% (95/112) were gram-negative bacteria. *Acinetobacter baumannii *(*A. Baumannii*), *Pseudomonas aeruginosa *(*P. aeruginosa*), and *Klebsiella pneumonia *(*K. pneumonia*) were the most frequently isolated MDRO strains. The logistic regression model and multifactorial analysis showed that long-term (≥ 7 days) antibiotic use (OR 6.901), history of tracheotomy (OR 4.458), and a low albumin level (< 40 g/L) (OR 2.749) were independent risk factors for the development of MDRO infection in patients in the rehabilitation ward (all *P* < 0.05). Gram-negative MRDOs were dominant in rehabilitation ward patients. Low albumin, history of a tracheostomy, and long-term use of antibiotics were independent risk factors for MRDO infection and are worthy of attention.

## Introduction

Multidrug-resistant organisms (MDROs) threaten the health patients under not only intensive care but also rehabilitation^1–3^. Patients admitted to the rehabilitation ward of our hospital (a 2000-bed general hospital) mainly have conditions affecting the central nervous system. Most of the patients are transferred from the Department of Neurosurgery and Critical Care Unit and have consciousness disorders, paralysis or impaired deglutition. Prolonged bed rest and long-term antibiotic use in these patients increase their vulnerability to bacterial infection^4^. Moreover, patients generally stay in rehabilitation centers for a long time and share rehabilitation training facilities. MDRO infections can easily occur and spread among these patients^5^.

A pilot study from Germany found that among MDROs, gram-negative, extended spectrum beta-lactamase (ESBL)-producing bacteria the highest prevalence rates among patients undergoing neurologic rehabilitation (10.2%) and geriatric rehabilitation (22.7%)^1^. Additional reports have found that the incidence of MDRO infections is increasing, and positive patients have a significantly higher burden than negative patients and attain fewer rehabilitative benefits^6,7^.

Recent studies from China investigated the profile and the antibiotic resistance patterns of MDROs in the intensive care unit (ICU) or among cancer patients^3,8^. However, there is a lack of systematic epidemiological investigations on nosocomial MDRO infections in rehabilitation wards of general hospitals in China. Therefore, the current study aims to investigate the microbiological profiles of and risk factors for MDRO infection in the rehabilitation ward of our hospital, which will provide valuable information for reducing the rate of MDRO infection in rehabilitation wards.

## Materials and methods

### Aim

We aim to investigate the prevalence of and risk factors for multidrug-resistant organism (MDRO) infection in the rehabilitation ward of a general hospital in Southwest China.

### Study design and settings

This is a descriptive retrospective observational cohort study and was conducted in neurorehabilitation wards of the second affiliated hospital of Chongqing Medical University. We retrospectively reviewed the medical charts of nosocomial infection patients hospitalized in the rehabilitation department of our hospital from June 1, 2016, to June 1, 2020.

### General information

**Figure 1:**
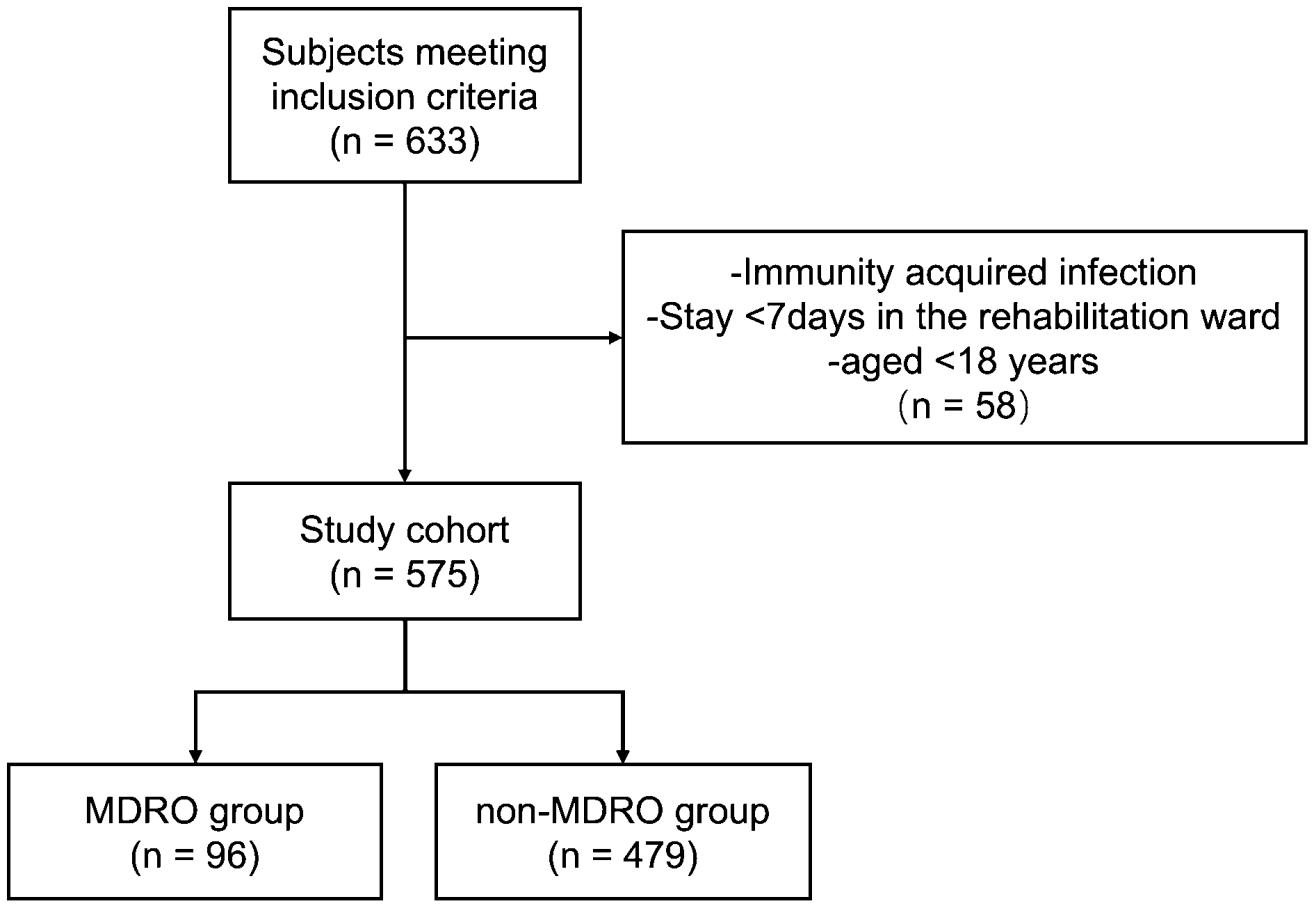
Patient enrollment flow chart.

A total of 575 patients were enrolled; 343 were males, and 232 were females. According to the presence or absence of MDRO infection, patients were divided into an MDRO group (n = 96) and a non-MDRO (n = 479) group. See Fig. 1.

The inclusion criteria were as follows: patients who (1) received treatment in the rehabilitation ward and had a hospital stay of more than 7 days; (2) had complete medical records; and (3) were aged ≥ 18 years.

The exclusion criteria were as follows: patients who (1) stayed less than 7 days in the rehabilitation ward; (2) showed evidence of existing infection on admission; (3) had incomplete medical records; and (4) were aged < 18 years. Duplicate isolates of the same bacteria isolated from the same patient were excluded from this study.

### Definitions

MDRO infection was defined by a physician as follows: (1) the isolated organism was non-susceptible to at least one agent in three or more antimicrobial categories^9,10^; (2) the clinical symptoms and signs and laboratory or radiology examination results indicated infection according to the descriptions of the Centers for Disease Control and Prevention^11^; and (3) a clear infection diagnosis was noted in the patient’s electronic medical chart. Nosocomial infection was defined as signs or symptoms of infection within > 48 h after hospital admission or at < 48 h after hospital discharge. If these criteria were not met, the infection was considered community acquired.

### Microbiological technique and bacterial susceptibility testing

Microbiology samples, such as sputum, urine, blood, feces and other secretions, were collected from patients with suspected bacterial infection. Swabs were collected for colonization screening but not for the diagnosis of MDRO infection^8^. Only the initial isolates were considered in our study and repeat isolates from the same sites were excluded. The specimens were processed according to the routine laboratory diagnostic protocol, which included morphological, biochemical and culture characteristics identifications. The purified isolates were identified with the use of VITEK^®^ 2 Compact (bioMérieux) and mass spectrometric approaches. Antimicrobial susceptibility testing was carried out using the microdilution broth method (minimum inhibitory concentration, MIC), and the results were determined according to the Performance Standards for Antimicrobial Susceptibility Testing (CLSI M100).

### Data collection

To identify clinical and epidemiological risk factors for infection by MDROs, data collection was carried out retrospectively with a monitoring questionnaire. The following clinical and epidemiological variables were analyzed: age, sex, consciousness state^12^, diabetes history, nutritional state, albumin level, hospitalization history, invasive procedure, biological and clinical signs of infection, date and site of infection, antibiotic usage, and specimen origin.

### Statistical methods

The data were entered into Microsoft Office Excel 2016, and the results were analyzed using the Statistical Package for the Social Sciences (SPSS 20.0, Chicago, USA). Count data (%) were compared by Fisher’s exact test or the chi-square test as appropriate. Fisher’s exact test was used if the expected count in any cell was less than five. Multivariate analyses were performed to evaluate the most important associations between risk factors and infection acquisition. Therefore, logistic multifactorial regression analysis was performed. The odds ratio (OR) and its corresponding 95% confidence interval (CI)^13^ for each factor was calculated to identify the level of association between the risk factor and the acquisition of MDRO^14^. A *P* value less than 0.05 indicates a significant difference.

### Ethics consideration

This study protocol was approved by the Ethics Committee of the Second Affiliated Hospital of Chongqing Medical University. All methods were performed in accordance with the relevant guidelines and regulations. As the data are anonymous, the requirement for informed consent was waived by the Ethics committee of the Second Affiliated Hospital of Chongqing Medical University.

## Results

### The origin of MDRO specimens

After excluded 58 patients from the 633 patients who meet the inclusion criteria, a total of 575 nosocomial infection patients were finally analyzed (Fig. 1). Meanwhile, there was a total of 3125 patients admitted in the neurorehabilitation center. After eliminating duplicate strains and colonization, 112 strains of MDRO were found positive from 96 patients. The MDRO test-positive rate was 16.70% (96/575). Different types of specimens were positive for MDROs, of which sputum accounted for 84.82% (95/112) and urine accounted for 9.82% (11/112). We also analyzed the distribution of infection types in patients of both groups and the result showed there was no significant differences (Supplementary Table 1). This result indicated that lung infections in patients in the rehabilitation ward were quite common (Table 1).

**Table 1:** Origins of specimens from patients with suspected bacterial infection.

Origin of specimen	No. of strains (n = 112)	Percentage (%)
Sputum	95	84.82
Urine	11	9.82
Feces	3	2.68
Secretions	2	1.79
Blood	1	0.89

### The composition of MDRO isolates

Among the 96 patients, 81 showed single MDRO infected and 14 showed 2 isolates of MDROs and 1 patient had 3 isolates of MDROs. And we analyzed the distribution of 95 strains of multidrug-resistant bacteria isolated from 79 pneumonia patients in the neurorehabilitation ward. The data showed that gram-negative, multidrug-resistant bacteria were the main isolates (95.79%, 91/95) responsible for lung infections in rehabilitation ward patients. Specifically, 91 strains were gram-negative, including 43 strains of *Acinetobacter baumannii *(*A. baumannii*) (45.26%, 43/95), 35 strains of *Pseudomonas aeruginosa *(*P. aeruginosa*) (36.84%, 35/95), 12 strains of *Klebsiella pneumoniae* (*K. pneumoniae*) (12.64%, 12/95)*, and* 1 strain of* Enterobacter cloacae* (*E. cloacae*) (1.05%, 1/95)*;* 4 g-positive methicillin-resistant *Staphylococcus aureus* (*MRSA*) accounted for 4.21% (4/95) of the total (Table 2). The distribution of the 112 MDRO isolations was shown in Supplementary Table 2.

**Table 2:** Compositions of multidrug-resistant pneumonitis pathogens in the rehabilitation ward.

Pathogen	No. of strains (95)	Percentage (%)
**Gram-negative bacteria**	91	95.79%
*A. baumannii*	43	45.26%
*P. aeruginosa*	35	36.84%
*K. pneumoniae*	12	12.64%
*E. cloacae*	1	1.05%
**Gram-positive bacteria**	4	4.21%
MRSA	4	4.21%

*A. baumannii*: Acinetobacter baumannii; *P. aeruginosa*:* Pseudomonas aeruginosa*;* K. pneumoniae: Klebsiella pneumoniae*;* E. cloacae: Enterobacter Cloacae*; MRSA: Methicillin-resistant staphylococcus aureus.

### Antibiotic resistance among MDRO isolates

Because the gram-negative MDROs from sputum took the main isolates, we next examined the antibiotic resistance profiles of the gram-negative MDRO strains in patients with pneumonitis. The results showed that gram-negative *A. baumannii* was 100% (43/43) resistant to ciprofloxacin and imipenem and 97.67% (42/43) resistant to piperacillin, piperacillin/tazobactam, ceftriaxone, ceftazidime, cefepime, aztreonam and meropenem. The *A. baumannii* isolates seemed susceptible to sulfonamide, cefoperazone/sulbactam and tigecycline, as the resistance rates were 44.19% (19/43), 18.6% (8/43) and 0% (0/43), respectively (Table 3). Similarly, the resistance of *K. pneumoniae* to several antibiotics was 100% (12/12), except tigecycline (0%, 0/12), cefoperazone/sulbactam (8.33%, 1/12), sulfonamide (25%, 3/12), and cefepime and piperacillin/tazobactam (both 91.67%, 11/12). In contrast, the resistance of *P. aeruginosa* to tigecycline, sulfonamide and ceftriaxone was 100% (35/35) (Table 3). The resistance of *P. aeruginosa* to other antibiotics was relatively low, for example, 34.92% (12/35) for piperacillin, 28.57% (10/35) for ceftazidime, 14.29% (5/35) for cefoperazone/sulbactam, and 2.86% (1/35) for amikacin (Table 3). As only one *E. cloacae* strain was isolated, calculating the percentage of antibiotic sensitivity seems not applicable.

**Table 3:** Antibiotic resistance results of gram-negative MDRO strains in patients with pneumonitis.

Antibiotics		*A. baumannii* (43)		*P. aeruginosa* (35)		*K. pneumoniae* (12)	
		Strains	Percentage	Strains	Percentage	Strains	Percentage
Beta-lactam antibiotics	Piperacillin	42	97.67	12	34.29	12	100
	Piperacillin/Tazobactam	42	97.67	13	37.14	11	91.67
	Ceftriaxone	42	97.67	35	100	12	100
	Ceftazidime	42	97.67	10	28.57	12	100
	Cefepime	42	97.67	11	31.43	11	91.67
	Cefoperazone/Sulbactams	8	18.60	5	14.29	1	8.33
	Aztreonam	42	97.67	13	37.14	11	100
	Meropenem	42	97.67	25	71.43	10	100
	Imipenem	43	100	29	82.86	10	100
Fluoroquinolones	Levofloxacin	34	79.07	15	42.86	11	100
	Ciprofloxacin	43	100	12	34.29	12	100
Others	Amikacin	30	69.77	1	2.86	10	100
	Sulfonamides	19	44.19	35	100	3	25
	Tigecycline	0	0	35	100	0	0

The results also supported the idea that *MRSA* was still sensitive to Tetracycline and Vancomycin in most cases (Supplementary Table 3).

### Association between patient characteristics and infection with MDROs

Finally, we analyzed the risk factors for MDRO-related infection in neurorehabilitation ward. The results showed that patients in the rehabilitation ward with a history of ICU stay, a low albumin level, a history of mechanical ventilation, presence of a tracheostomy, an indwelling urinary catheter, indwelling nasogastric intubation, and long-term antibiotic use and those who were male were more likely to develop MDRO infection than patients with nosocomial infection but not infected with an MDRO (Table 4). However, further analysis of the above risk factors in the logistic regression analysis revealed that a low albumin level, presence of a tracheostomy, and long-term antibiotic use were the only independent risk factors (*P* < 0.05) for the development of MDRO-related infectious in patients in the neurorehabilitation ward (Table 5).

**Table 4:** Risk factors for MDRO-related infection in neurorehabilitation ward.

Risk factors		Total (N = 575)	MDRO (N1 = 96, %)	non-MDRO (N2 = 479, %)	Percentage (N1/N, %)	*χ*^2^	P
Age	< 65	259	51, 53.13	208, 43.42	19.69	3.040	0.081
	≥ 65	316	45, 46.87	271, 56.58	14.24		
Sex	Male	343	72, 75.00	271, 56.58	20.99	11.276	0.001
	Female	232	24, 25.00	208, 43.42	10.34		
Awareness^**a**^	Con	433	66, 68.75	367, 76.62	15.24	2.662	0.103
	Uncon	142	30, 31.25	112, 23.38	21.12		
Diabetes	Yes	122	26, 27.08	96, 20.04	21.31	2.372	0.124
	No	453	70, 22.92	383, 79.96	15.45		
Albumin level	≥ 40 g	409	45, 46.88	364, 75.99	11.00	33.016	0.000
	< 40 g	166	51, 53.12	115, 24.01	30.72		
ICU history	Yes	98	30, 33.33	68, 14.20	30.61	16.450	0.000
	No	477	66, 66.67	411, 85.80	4.23		
Mechanical Ventilation	Yes	90	30, 33.33	60, 12.53	33.33	21.237	0.000
	No	485	66, 66.67	419, 87.47	13.60		
Tracheotomy	Yes	35	24, 25.00	11, 2.30	68.57	72.111	0.000
	No	540	72, 75.00	468, 97.70	13.33		
Nasogastric tube	Yes	179	82, 85.42	97, 20.25	45.81	158.406	0.000
	No	396	14, 14.58	382, 79.75	3.53		
Indwelling Catheter	Yes	220	71, 73.96	149, 31.11	32.27	62.167	0.000
	No	355	25, 26.04	330, 68.89	7.04		
Long-term Antibiotic use	< 7 days	347	7, 7.29	340, 70.98	2.01	135.565	0.000
	≥ 7 days	228	89, 92.71	139, 29.02	39.03		

^a^The awareness was assessed using CRS-R and GCS. *CRS-R* Coma Recovery Scale—Revised, *GCS* Glasgow Coma Scale, *con* conscious, *uncon* nonconscious, *ICU* intensive care unit.

**Table 5:** Multifactorial logistic analysis of risk factors for MDRO-related infection

Risk factor	*β*	SE	Wald	*P*	OR	95% CI
Low albumin level (< 40 g)	1.011	0.275	13.565	0.000	2.749	1.605–4.708
Tracheotomy	1.495	0.448	11.142	0.001	4.458	1.854–10.724
Long-term antibiotic usage (≥ 7 days)	1.932	0.308	39.214	0.000	6.901	3.770–12.632

## Discussion

Antibiotic resistance is an ongoing major public health challenge worldwide. Studies have noted that the prevalence rates of risk factors for MDRO infection have increased in recent years^13,15,16^. Therefore, MDRO infections have been regarded as a global health priority. As the study was conducted in a neurorehabilitation center, patients with acquired brain injury comprised the main inpatient population in our study. Usually, transfer to the rehabilitation ward indicates that the patient’s condition is relatively stable. However, neurologic injury patients (e.g., acquired brain injury patients) have an increased risk of infection due to injury-related immune deficits^17^, the accumulation of comorbid conditions^18^, prolonged hospital stay, and severe functional and cognitive dysfunctions that increase dependence on caregivers^19^. Additionally, the interactive nature of rehabilitation wards, such as shared therapeutic facilities and close contact with therapists/nurses, provides opportunities for communicable diseases to spread^20,21^.

Our investigation revealed that the prevalence of MDRO infection among nosocomial infection patients in the neurorehabilitation ward was 16.70%. Specifically, 95 MDRO strains were detected in sputum, of which 95.79% were gram-negative bacteria, with *A. baumannii* (accounting for 45.26%), *P. aeruginosa* (accounting for 36.84%), and *K. pneumonia* (accounting for 12.64%) being the most prevalent. In contrast, a previous review study reported that among MDROs, ESBL-producing *Enterobacterales* (71.6%) and carbapenem-resistant (CR) *Enterobacterales* (6.9%) were the most prevalent in Asia, while multidrug-resistant *P. aeruginosa* (5.4%), multidrug-resistant *A. baumannii* (15.0%), and *C. difficile* (26.1%) were the most prevalent in North America^13,22^. Therefore, different study populations may have different MDRO prevalence, patient populations, and antibiotic susceptibility profiles. Only 4 strains (4.21%) of *MRSA* were detected in our study. This is not surprising, as the prevalence of *MRSA* showed a markedly decreasing trend from 69.0% in 2005 to 35.3% in 2017 based on data from the China Antimicrobial Surveillance Network (CHINET)^23^. Additionally, in the study by Heudorf and colleagues, the *MRSA* prevalence was only 1.3% among patients undergoing neurologic rehabilitation^24^. Another reason might be because of the specimen composition. In this study, sputum samples accounted for over 90% (gram-negative MDRO are more likely to appear in sputum samples), while *Staphylococcus* (e.g. *MRSA*) will more likely appear in samples from skin and soft tissue infection, so it accounts for a very low proportion^25^.

It should be noted that antimicrobial susceptibility varied among the gram-negative MDRO isolates. For example, *A. baumannii* was resistant to mainly piperacillin, cephalosporin, and carbapenems (Meropenem and Imipenem) but was susceptible to cefoperazone/sulbactams, tigecycline and sulfonamides. In contrast, *P. aeruginosa* was 100% resistant to tigecycline and sulfonamides and over 70% resistant to carbapenems. We also found that *K. pneumoniae* was 100% resistant to carbapenems, aztreonam, amikacin, levofloxacin, ceftriaxone, and piperacillin. In recent years, carbapenem-resistant gram-negative bacteria, especially multidrug-resistant *K. pneumoniae*, have emerged as a new threat causing both nosocomial and community-acquired infections worldwide. In a study in cancer patients, researchers found that the isolated gram-negative MDROs were primarily sensitive to meropenem, imipenem, and amikacin, while they were primarily resistant to aztreonam, cephalosporins, and fluoroquinolones^8^. This might be partly attributed to the different pathological characteristics of patients in neurorehabilitation and oncology centers. The proportion of *K. pneumoniae* with carbapenem resistance has increased rapidly in not only undeveloped areas but Europe and North America^15^. Since carbapenems are often considered last-resort antibiotics for severe *K. pneumoniae* infection, treatment will become difficult if carbapenem resistance develops. Zhu et al. found that exposure to carbapenems is one of the main risk factors (OR 4.16) for carbapenem-resistant *K. pneumoniae* infection^15^. Therefore, clinical expertise suggests that restricting the use of carbapenems is helpful in reducing the development of MDROs.

The analysis of risk factors revealed that patients in the rehabilitation ward with a history of ICU stay, a low albumin level, a history of mechanical ventilation, presence of a tracheostomy, an indwelling urinary catheter, indwelling nasogastric intubation, and long-term antibiotic use as well as those who were male were more likely to develop MDRO infection. Logistic regression further confirmed that a low albumin level, presence of a tracheostomy, and long-term antibiotic use were independent risk factors. Despite warnings regarding overuse, antibiotics are overprescribed worldwide. Based on our results, long-term exposure (> 7 days) to antibiotics is more likely to induce multidrug resistance in rehabilitation patients than short-term exposure. This is consistent with Hanna Renk’s study in ICU patients^26^. The reason might be that antibiotics remove antibiotic-sensitive competitors, leaving resistant bacteria behind to reproduce as a result of natural selection. Limiting antibiotic therapy when feasible and optimizing antibiotic use duration have the potential to enhance patient care while preventing multidrug-resistant infections^27^.

Infection and malnutrition have always been intricately linked^28^. Albumin levels lower than 40 g/L were recognized as a risk factor for MDRO infection in our study. This is inconsistent with the results of Schoevaerdts’s study in older patients^29^. As a result of inadequate protein and caloric intake in stroke patients with dysphagia^30^, hypoalbuminemia is quite common in the rehabilitation ward. Early studies noted that serum albumin plays a role in antiplatelet aggregation activity and antioxidant and anti-inflammatory processes^31^. Additionally, low albumin levels impair the immune system. A recent study reported that human serum albumin alters the expression of specific genes that promote the survival and persistence of *A. baumannii*^32^. Moreover, hypoalbuminemia increases the apparent total volume of distribution (V (d)) and clearance^33^ of time-dependent antibacterial, which may result in a failure to achieve pharmacodynamic targets^34^. Therefore, a maintained nutritional supply is of utmost importance to ensure adequate albumin production, especially in dysphagia patients in the rehabilitation ward.

A recent meta-analysis showed that an endotracheal tracheostomy obviously increased the risk of multidrug-resistant bacterial infection^35^. This was also confirmed in our study, as MDRO-related infection was 4 times more common in rehabilitation patients with a tracheostomy than in those without. Previous studies demonstrated that patients who had a tracheostomy were more likely to develop lung infection by *P. aeruginosa* and carbapenem-resistant *Klebsiella*, both of which are common MDROs^36,37^. The presence of a tracheostomy tube not only increases flow resistance and breathing effort but also impairs the heating and humidification of inspired air^38^. Consequently, ciliary function is damaged, and respiratory infection recurs. Decannulation is a milestone of physical recovery for patients after being transferred to the rehabilitation center^39^. Based on previous guidelines and experience, the sooner patients are decannulated, the less their risks for acquiring pneumonia and MDRO infection are^35,40^. Zivi et al. demonstrated that early mobilization plays a critical role in reducing the time to decannulation^41^. Moreover, sufficient respiratory muscle performance, cough efficacy, swallowing status, and consciousness supports the cannula removal process in most patients^38,39^. Therefore, comprehensive rehabilitation strategies, including secretion management, respiratory muscle training, airway clearance techniques, swallowing exercises and pharyngeal electrical stimulation, have been suggested for tracheotomized patients in clinical practice^42–44^.

In summary, the prevalence of nosocomial infection due to a MDRO was relatively high in rehabilitation patients in our study. The most frequently isolated pathogens were gram-negative *A. baumannii*, *P. aeruginosa*, and *K. pneumonia*. The limitation of this study was that data were obtained from only one hospital; thus, there is a possibility that other types of pathogens were neglected. We suggest effective hygiene management in rehabilitation wards, and physicians should consider the epidemiological characteristics of local resistance patterns when initiating antimicrobial treatment.

## Supplementary Information

Supplementary Tables.

## Data Availability

The datasets generated and/or analyzed during the current study are available from the corresponding author on reasonable request.
