# Supplementary material for: Gram-negative multidrug-resistant organisms were dominant in neurorehabilitation ward patients in a general hospital in southwest China
Source: Sci Rep. 2022 Jun 30;12:11087. doi: 10.1038/s41598-022-15397-y (PMC9246850; doi:10.1038/s41598-022-15397-y)
Supplement: Supplementary file 1 — Supplementary Tables. [file 41598_2022_15397_MOESM1_ESM.docx]

**Supplement Table 1** Distribution of infection types in patients

| Type of infection | Total  (N=575) | MDRO  (N1=96) | non-MDRO  (N2=479) | *χ^2^*, *P* value |
| --- | --- | --- | --- | --- |
| Lung infection (LI) | 427 | 79 | 348 | 4.049, *0.132* |
| Urine tract infection (UTI) | 88 | 11 | 77 |  |
| Other infection | 60 | 6 | 54 |  |

**Supplement Table 2** Compositions of MDRO isolations in the rehabilitation ward

| Pathogen | No. of strains (112) | Percentage (%) |
| --- | --- | --- |
| **Gram-negative bacteria** | **101** | **90.18** |
| *A. baumannii* | 45 | 40.18 |
| *P. aeruginosa* | 36 | 32.14 |
| *K. pneumoniae* | 14 | 12.50 |
| *E. cloacae* | 4 | 3.57 |
| *C. freundii* | 2 | 1.79 |
| **Gram-positive bacteria** | **11** | **9.82** |
| *MRSA* | 8 | 7.14 |
| *C. difficile* | 3 | 2.68 |

**Supplement Table 3** Antimicrobial resistance of MDROs cultured from 11 urine samples.

| Strain | Source of specimen | Resistance of Antibiotics | | | | | | | | |
| --- | --- | --- | --- | --- | --- | --- | --- | --- | --- | --- |
| MRSA |  | Penicillin G | Oxacillin | Ampicillin | Erythromycin | Levofloxacin | Gentamicin | Clindamycin | Tetracycline | Vancomycin |
|  | Secretion | **+** | **+** | **+** | **-** | **+** | **-** | **-** | **-** | **-** |
|  | Sputum | **+** | **+** | **+** | **+** | **+** | **-** | **+** | **-** | **-** |
|  | Urine | **+** | **+** | **+** | **-** | **-** | **-** | **-** | **-** | **-** |
|  | Sputum | **+** | **+** | **+** | **+** | **-** | **+** | **+** | **-** | **-** |
|  | Sputum | **+** | **+** | **+** | **+** | **+** | **+** | **+** | **-** | **-** |
|  | Urine | **+** | **+** | **+** | **+** | **+** | **+** | **+** | **-** | **-** |
|  | Sputum | **+** | **+** | **+** | **+** | **-** | **-** | **+** | **-** | **-** |
|  | Secretion | **+** | **+** | **+** | **+** | **-** | **-** | **+** | **-** | **-** |

+ represent drug resistance; - represent drug sensitive
